# Insights on the role of l-lactate as a signaling molecule in skin aging

**DOI:** 10.1007/s10522-023-10018-1

**Published:** 2023-01-28

**Authors:** Salvatore Chirumbolo, Dario Bertossi, Pierre Magistretti

**Affiliations:** 1 Department of Neurosciences, Biomedicine and Movement Sciences, Unit of Human Anatomy, University of Verona, Strada Le Grazie 8, 37134 Verona, Italy; 2 Department of Surgery, Dentistry, Paediatrics and Gynaecology-Unit of Maxillo-Facial Surgery, University of Verona, Verona, Italy; 3 Biological and Environmental Sciences and Engineering Division, King Abdullah University of Science and Technology (KAUST), Thuwal, 23955 Saudi Arabia

**Keywords:** l-lactate, Aging, Mito-hormesis, Skin, Redox signaling

## Abstract

l-lactate is a catabolite from the anaerobic metabolism of glucose, which plays a paramount role as a signaling molecule in various steps of the cell survival. Its activity, as a master tuner of many mechanisms underlying the aging process, for example in the skin, is still presumptive, however its crucial position in the complex cross-talk between mitochondria and the process of cell survival, should suggest that l-lactate may be not a simple waste product but a fine regulator of the aging/survival machinery, probably via mito-hormesis. Actually, emerging evidence is highlighting that ROS are crucial in the signaling of skin health, including mechanisms underlying wound repair, renewal and aging. The ROS, including superoxide anion, hydrogen peroxide, and nitric oxide, play both beneficial and detrimental roles depending upon their levels and cellular microenvironment. Physiological ROS levels are essential for cutaneous health and the wound repair process. Aberrant redox signaling activity drives chronic skin disease in elderly. On the contrary, impaired redox modulation, due to enhanced ROS generation and/or reduced levels of antioxidant defense, suppresses wound healing via promoting lymphatic/vascular endothelial cell apoptosis and death. This review tries to elucidate this issue.

## Introduction: l-lactate as a signaling molecule

l-lactate, is known also as l-lactic acid, i.e., an α-hydroxyl acid and is a ubiquitous molecule coming from the reduction of l-pyruvate into l-lactate by the l-lactate dehydrogenase (LDH, E.C. 1.1.1.27), to produce NAD + from NADH in the Embden–Meyerhof–Parnas’s pathway. l-lactate is commonly considered an apparent useless byproduct of glycolysis (Rogatzki et al. 2015). Actually, since many years, l-lactate has been mistakenly described as simply a toxic remnant of the anaerobic metabolism, despite recent evidence would suggest a crucial role for l-lactate in the cell biology (Ratter et al. 2018; Philp et al. 2005; Brooks 2020; Manosalva et al. 2022).

This l-enantiomer can be formed even under aerobic conditions with a specific signaling function (Brooks 2020; Baltazar et al. 2020; Tauffenberger et al. 2019). As outlined in this overview, l-lactate serves as a master regulator of many complex pathways regulating crucial cell functions. Its ability to exert fundamental signaling functions, sets l-lactate within the major crucial pathways of cell survival (Lee 2021).

However, despite its crucial role in many steps of the metabolic check-point leading to cell survival and differentiation, a relatively scant literature exists about l-lactate as an on/off switcher of major intracellular pathways, involved in aging and survival of skin. A survey on Pubmed/Medline with the MESH term “lactate AND aging AND signaling” retrieved 283 releases, yet only 11 if the term “skin” is added. Despite the wide use of l-lactate in many cosmetic formulations, the role of this molecule in regulating skin function and skin senescence is yet far to be fully accomplished (Huang et al. 2020).

The modulating activity of l-lactate is closely linked with its ability to work as a signaling factor in crucial steps of the cell machinery. Usually, the evolution has selected small, ubiquitous and pleiotropic molecules to act as signaling molecules, for example reactive oxygen species (ROS) (D'Autréaux and Toledano 2007), nitric oxide (NO) (Tuteja et al. 2004), adenosine from ATP (Eltzschig 2013), carbon monoxide (Mann 2010) and so forth. In particular, carbon monoxide (CO) is an endogenously derived gas formed from the breakdown of haeme by the enzyme haeme oxygenase 1 (HO-1). Although long considered an insignificant and potentially toxic waste product of haeme catabolism, CO is now recognized as a key signaling molecule that regulates numerous metabolic functions including the many cytoprotective, antioxidant, and anti-inflammatory abilities (Durante et al. 2006; Kim et al. 2006).

## l-lactate role in the aging process and the role of mitochondria

In recent years, despite the role of l-lactate has been widely associated, as a biomarker, with the noxious activity of stressors, xenobiotics and endogenous toxicants (Seheult et al. 2017; Schmidt and Karlson-Stiber 2008; Manojlović and Erčulj 2019), evidence was reported about a surprising beneficial activity of this α-hydroxyl acid. For example, l-lactate protects skin fibroblasts from mitochondria aging-related dysfunction, via a process known as “mito-hormesis” (Zelenka et al. 2015).

Mito-hormesis (Barzegari et al. 2022; Bárcena et al. 2018; Bordon 2021) is a modulating process, involving mitochondria and the mitochondria-associated membrane (MAM) system (van Vliet and Agostinis 2018; van Vliet et al. 2014), where a “mild” stress induction can lead to a persistent cellular adaptation to stressors. This adaptation enables the cell to prevent damage, enhance its survival response and activate rejuvenation and/or biogenesis processes, in order to protect the cell from apoptosis and mitochondria from dysfunction.

As a matter of fact, mitochondria may reduce the impact of reactive oxygen species (ROS)-mediated damage by uncoupling the oxidative phosphorylation via the uncoupling protein-1 but also by modulating the level of ROS as signaling molecules by means of the mito-hormesis (Atayik and Çakatay 2022). The role of l-lactate in this process is yet under investigation. Mito-hormesis can be considered as an adaptive stress response via a kind of mito-nuclear signaling, acting in order to enhance cell survival and stress resistance, for example by inducing the release of factors such as fibroblast growth factor 21 (FGF21) and growth and differentiation factor 15 (GDF15), two fundamental stress-triggered mitokines (Klaus and Ost 2020), to cite l-lactate role in the skin.

If l-lactate has a beneficial role on cell survival via a mito-hormetic mechanism, then it should lead to a fundamental modulation of the ROS signaling. In this context, l-lactate regulation of the stress response, and hence of cell survival and differentiation, appears particularly intriguing also to elucidate the aging process, if l-lactate is at the same time a waste product and a major signaling molecule.

More generally, it is tempting to speculate that one of the major roles of highly ubiquitous and largely pleiotropic molecules, such as CO and l-lactate, usually coming from the complex metabolic network of signaling and enzymatic pathways, is to be widely used as fine regulators. Therefore, as occurring with ROS, ATP, adenosine, calcium ions and so on, l-lactate too, should be re-interpreted as a signaling molecule with fundamental modulatory actions (Coggan et al. 2022; Veloz Castillo et al. 2021).

During the aging process, l-lactate exerts fundamental actions in the brain, as it has been recently considered also a biomarker of brain senescence, where the ratio LDH isoenzymes A/B may be altered, though controversial opinions yet remain (Ross et al. 2010; Datta and Chakrabarti 2018). In a recent mitochondrial theory of aging, dating back to several years, mitochondria dysfunction and damage in the mitochondrial DNA (mtDNA), are collectively included in the causative panoply of factors leading to the aging process of cells and tissues (Harman 1972; Larsson 2010). This might elucidate the possible role of l-lactate in the subsequent impairment of mitochondria activity, leading the cell to switch mainly towards aerobic mode of fermentation, so regulating fundamental pathways in the survival machinery.

Prematurely aged mice with mutated mtDNA, have mitochondria shifting aerobic metabolism to a glycolytic pathway (Ross et al. 2010). The intrinsic meaning of this mechanism is still under investigation, yet it involves l-lactate, and suggests a role for this molecule as a master regulator of mitochondria biology (Ross et al. 2010). As a matter of fact, the role of mitochondria in aging has been widely confirmed in the most recent literature (Sun et al. 2016; Srivastava 2017; Jang et al. 2018). In this perspective, l-lactate may be a leading factor in the adjusting of mitochondria physiology (Van Hall 2000; Levasseur et al. 2006). Interestingly, recent data reported that an intermittent treatment of skin fibroblasts with l-lactate induced a mild inhibition of the mitochondrial aerobic process via the respiratory chain (mito-hormesis), causing production of hydrogen peroxide, phosphorylation of AMPK and activation of the mitochondria biogenesis via PGC-1α, so activating the cellular endowment of survival genes and enzymes and finally acting as a skin protective factor (Ross et al. 2010). Hydrogen peroxide is involved in the intracellular signaling of ROS, acting as a mediator of several physiological processes such as cell differentiation and proliferation, cellular metabolism, survival, and immune response (Di Marzo et al. 2018).

This evidence should suggest that moderate levels of l-lactate, for example during muscular exercise, may have beneficial effects, allowing to consider l-lactate as a signaling molecule in the complex role exerted by mitochondria in the aging process, using sustained, moderate exercise (Musci et al. 2019; Merry and Ristow 2016).

However, the role of l-lactate in muscles might include a much wider complex of signaling factors and pathways, which encourages researchers to define its task in a more systemic landscape than the simplest biochemical machinery associated with mitochondria. Past reports, showing a beneficial action of topical l-lactate on skin biology, might have a possible elucidation by the most recent research in the field (Smith 1996; Tran et al. 2014; Huang et al. 2020).

l-lactate may be closely related to aging as, in animal models, the level of this glycolytic byproduct rapidly increases in the senescent phenotype (Datta and Chakrabarti 2018; Wallace et al. 2020).

Senescent cells exhibit higher glycolytic activity and lactate production than youngest cells, alongside with an enhanced expression of lactate dehydrogenase A as well as increases in tricarboxylic acid cycle activity and mitochondrial respiration. The latter is likely due to the reduced expression of pyruvate dehydrogenase kinases (PDHKs) in senescent cells, which may lead to increased activity of the pyruvate dehydrogenase complex (Stabenow et al. 2022).

At least in murine models, the circadian rhythms of lactate in aged C57BL/6N male mice (19 months) appear slightly phase-advanced respect to younger animals, affecting the metabolic activity of the prefrontal cortex in the brain (7 months). A possible reason can be hypothesized by observing the reduction in GLUT-1 receptors alongside with the aging process (Wallace et al. 2020). If aging modulates the systemic involvement of l-lactate in the individual’s metabolic homeostasis, then l-lactate should have a fundamental role even in the human physiology.

Actually, circadian rhythms of l-lactate were reported also in humans, particularly during physical exercise (Forsyth and Reilly 2004; Reilly and Waterhouse 2009), and furthermore recent evidence suggests that a cross talk between metabolism and circadian rhythms can be described (Reinke and Asher 2019).

At a cellular level, where cells should have their own circadian oscillators, an interplay dynamic between circadian clocks and the mammalian target of rapamycin (mTOR) pathway was reported (Guerrero-Morín and Santillán 2020). This perspective suggests that the role of l-lactate as a master regulator of aging must be much more systemic that expected.

So far, the linkage between l-lactate, aging and redox modulation, is overshadowed by a huge crowd of molecular participants in the highly complex milieu describing the fundamental role of mitochondria in the cell survival. This might explain why scientific literature is particularly scant in gathering reports showing the role of l-lactate, not exclusively as a catabolite, despite some recent reports on the topic of l-lactate as a signaling molecule (Table 1). In this review we attempt to elucidate the role of l-lactate as a signaling and regulatory molecule in the aging process, focusing particularly on the senescence process of the skin.

**Table 1:** Skin aging and the role of l-lactate as a signalling molecule

Type of study	Evidence reported	Action	Role as signaling molecule	References
In vitro rat skin fibroblasts	Activity and expression of AMPK, master regulation of stress response Mitochondria biogenesis l-lactate intermittency protection of mitochondrial dysfunction Skin aging and senescence (via PGC-1α and autophagy)	⇑ ⇑ ⇑ ⇓	l-lactate elicits ROS as signaling molecules via mitohormesis	Zelenka et al. (2015)
Review	HIF-1α stabilization Promotion of IFN-γ and immune regulation	⇑ ⇑	l-lactate in HIF-1α/PHD via ROS	Lee (2021)
Review	Promotion of brain function in aging	⇑	l-lactate interacts with GPR81/HCA1 l-lactate signaling via ROS	Mosienko et al. (2015), Cai et al. (2022)
HaCaT cells (keratinocytes)	l-lactate from Lactobacilli action on senescent wound healing l-lactate from Lactobacilli action on keratinocytes migration l-lactate from Lactobacilli action on keratinocytes replication l-lactate from Lactobacilli action on inflammation	⇑ ⇑ ⇑ ⇓	l-lactate as signaling molecule in immunity	Brandi et al. (2020)
Review	Histone lactylation in gene expression. A 'lactate clock' of endogenous origin in M1 macrophages challenged with microbes turns on gene expression to promote homeostasis	⟺	l-lactate as signaling molecule in lactylation	Zhang et al. (2019)
Observational study	Epidermal and dermal firmness and thickness Lines and wrinkles	⇑ ⇓	l-lactate as a signaling molecule in ROS biology	Smith (1996)
Review	Maintenance of long term potentiation in neurons from astrocyte-derived l-lactate Brain plasticity and synaptogenesis Adaptation of brain caused by exercise	⇑ ⇑ ⇑	l-lactate as a signaling factor in neuronal activity	Huang et al. (2021)
Review	l-lactate as a signaling molecule in regulating exercise	⇑	l-lactate as a signaling molecule in ROS biology	Nalbandian and Takeda (2016)
Review	Brain plasticity and synaptogenesis Adaptation of brain caused by exercise	⇑ ⇑	l-lactate as signaling molecule likewise BDNF	Müller et al. (2020)
Review	l-lactate in metabolic regulation	⟺	l-lactate as a signaling molecule in ROS biology	Pellerin et al. (2022), Wu et al. (2022)

*BDNF* brain-derived neurotropic factor, *HIF-1α* hypoxia inducible factor 1-alpha, *IFN-γ* interferon gamma, *PHD* prolyl hydroxylase, *ROS* reactive oxygen species, ⇑ activation (promotion), ⇓ inhibition (reduction), ⟺ modulation

## Insights about l-lactate as a signaling and modulatory molecule

### Role of ROS

Skin aging, health and disease are closely intertwined with mitochondria biology (Sreedhar et al. 2020). As the epidermis is a highly self-renewing tissue, the role of mitochondria may be therefore crucial (Zhang et al. 2018; Stout and Birch-Machin 2019). Furthermore, in this context, ROS play also a modulatory role in differentiating numerous cell lineages via downstream pathways such as C/EBP, β-catenin and Notch, even promoting differentiation in murine embryonic stem cells and the induction of both pluripotent stem cells and multipotent stem cells of epithelial origin (Lisowski et al. 2018). In conditional knock out mice, for the expression of the mitochondria transcription factor A (TFAM), some authors reported a high mortality rate caused by the absence of a correct functionality in the epithelial barrier and primary keratinocytes from these laboratory animals were unable to differentiate in vitro (Hamanaka et al. 2013). Actually, TFAM is involved in leading the replication of mitochondrial DNA and those cells lacking TFAM have impaired oxidative phosphorylation and ROS production (Hamanaka et al. 2013). This perspective strongly suggests that ROS are crucial in the signaling of skin health, including mechanisms underlying wound repair, renewal and aging. The outstanding role of ROS, as signaling molecules for skin health and aging, should be therefore reappraised (Ndiaye et al. 2014; Dunnill et al. 2017; Gauron et al. 2013).

ROS are major contributors in skin renewal, stem cell biology and keratinocyte differentiation. Alongside with ROS, it is presumable that l-lactate might play a role in skin aging and skin renewal, not so differentially from the signaling function exerted by ROS.

In this perspective, the relationship between ROS and l-lactate should be better highlighted.

A recent paper by Tauffenberger et al. reported that l-lactate, as well as l-pyruvate, is able to activate a stress response mechanism by eliciting a hormetic response of ROS, so activating the Nrf2/Keap1/ARE system and the unfolded protein response (UPR) (Tauffenberger et al. 2019). The question one might raise is whether l-lactate would act as a mild stressor, such as plant flavonoids, phytochemicals and other xenobiotics, for example, or if this catabolite has a much higher importance, in the cell economy.

Certainly, as l-lactate is the major product of glycolysis, its role cannot be compared to the occasional beneficial activity of a xenobiotic, due to hormetic mechanisms. Interestingly, aryl hydrocarbon receptors (AhRs), which act as transcription factors, modulate the genetic expression of several enzymes generating uridine monophosphate, alongside with LDHA, therefore controlling l-lactate production (Lafita-Navarro et al. 2020). The close relationship of xenobiotic biology via AhRs and l-lactate levels is suggestive for a signaling role of l-lactate in the oxidative stress response.

Impaired redox modulation of signaling pathways is a leading causative factor of cellular senescence.

### Role of other factors: autophagy and apoptosis

It is now well established that a subtle balancing of mitochondria activity via signaling molecules such as ROS and the fine regulation of biochemical pathways leading to the control of cell survival, is the main mechanism causing aging. Probably, aging is not merely an end-oriented process but the breakage of minute intracellular equilibria, which then may lead to an irreversible development and pathophysiology (Liguori et al. 2018; Kruk et al. 2019; Kitada and Koya 2021). The role of l-lactate in autophagy, for example, can provide further insights about its ability to modulate important signaling pathways in cell biology. It is well known that LDH-B and LDH-A isoenzymes control autophagy (Brisson et al. 2016; Das et al. 2019).

Autophagy is a fundamental process to maintain longevity (Barbosa et al. 2019) and recent data reported that l-lactate regulates autophagy, via the ERK1/2/m-TOR/p-70S6K pathway (Nikooie et al. 2021). Even this role in autophagy might have a hormetic cause, as the same l-lactate has an inhibitory action on autophagy when cells have lost their stress responsivity, such as in tumors (Matsuo et al. 2019). Again, in this perspective, l-lactate should exert a fundamental regulatory activity even on apoptosis (Go et al. 2021). At least on HepG2 cell lines, the increase in the extracellular lactate-to-pyruvate ratio has the ability to reduce the cytosol NADH/NAD^+^ redox state and inhibiting stress-induced apoptosis (Go et al. 2021). Moreover, high extracellular l-lactate inhibits the intrinsic apoptotic pathway by reducing the activation of JNK and Bax (Go et al. 2021).

The effect on the apoptotic pathway is of the utmost importance for aging, as reported by past reports regarding skin senescence (Haake et al. 1998; Zhang and Herman 2002; Tower 2015), but it is more presumably autophagy that mainly controls the process of skin aging (Eckhart et al. 2019). In this perspective, it is tempting to speculate if l-lactate might have a role in skin protection or repair, due to its modulatory activity on these mechanisms.

### l-lactate in skin aging

First intriguing evidence is that l-lactate is able to stimulate both the expression of CD44 and hyaluronan in H8 27 human dermal fibroblasts (Stern et al. 2002). The authors reported that the Warburg-like effect, leading to an increase in l-lactate, was probably the consequence of a blood and oxygen reduction in a wound repair mechanism. As a matter of fact, the incubation of H8 27 human fibroblasts with l-lactate enhanced the expression of the hyaluronan receptor CD44 and the production of hyaluronic acid (Stern et al. 2002). During wound repair, l-lactate accumulates and l-lactate itself works as an inducer of ROS to promote dermal fibroblasts growth, via the requirement of iron and hydrogen peroxide (Wagner et al. 2004). Moreover, it is well known that aging has a detrimental effect on skin fibroblasts, both reducing their growth and altering the expression of a wide plethora of collagen, metallothionein, interleukin, caspase and sirtuin genes (Lago and Puzzi 2019). Therefore, l-lactate may even exhibit an anti-aging role (Tran et al. 2014). Finally, l-lactate promotes the shift from the mitochondrial oxidative phosphorylation (OXPHOS) to glycolysis via HIF-1α and stabilizes the activity of the same hypoxic factor HIF-1α, probably in order to support the signaling function of ROS in human fibroblasts (Kozlov et al. 2020).

The relationship between ROS, mitochondria and l-lactate is of utmost importance for the cell survival, involving glucose metabolism as a switching control (Liemburg-Apers et al. 2015). A possible vicious cycle ROS-glycolysis may lead to cell death when ROS signaling is impaired (Liemburg-Apers et al. 2015).

ROS signaling is ruled by H_2_O_2_, (Forman et al. 2010) and hydrogen peroxide is crucial for a complex network of such biochemical hubs linking cell metabolism with the aging process (Roger et al. 2020). So, cellular aging may be the puzzling resultant of a complex interplay between ROS and l-lactate, via H_2_O_2_ scavenging enzymes such as peroxiredoxins (Roger et al. 2020).

The mechanisms of skin aging have been thoroughly reviewed (Jenkins 2002; Kohl et al. 2011; Zhang and Duan 2018). A key mechanism of skin aging is the induction of ROS and metalloproteinases (Kohl et al. 2011). These components are the major alarming signals of tissue and cell damage and a powerful biomarker of critically illness circumstances.

A close relationship between metalloproteinase-9 (MMP9) and tissue inhibitor of matrix metalloproteinase-1 (TIMP1) with l-lactate in plasma of critically ill patients, has been recently reported (Duda et al. 2020). Aging involves a thorough remodeling of tissues making the skin and matrix metalloproteinases are fundamental actors in the complex turnover of the extracellular matrix (ECM) and of cell composition in the connective tissue (Freitas-Rodríguez et al. 2017). It is presumable that l-lactate might exert a major activity in this complex scenario. Furthermore, in an effort to elucidate which kind of functional relationship l-lactate engages with the complex intracellular milieu of factors regulating ROS signaling and aging, where glucose metabolism might be the major key.

### Role of glucose

A past paper by Park et al. highlighted the evidence that a sustained hyperglycemic state, such as occurring during type 2 diabetes, induces an impaired skin barrier state, probably because skin homeostasis is delayed (Park et al. 2011). High glucose induces alterations in sirtuins, causing also a rapid aging in endothelial cells via forkhead transcription factors (FOXO) and p300 regulated pathway (Mortuza et al. 2013). Actually, in diabetic animals, endothelia showed signs of senescence, senescence associated β-gal (SA-β-gal) expression, reduction in sirtuins 1–7 and in FOXO1 DNA binding ability (Mortuza et al. 2013). If excess of glucose is a leading factor of senescence induction (Liu et al. 2020a; Zhang et al. 2017; Danby 2010; Yin et al. 2021), particularly for mesenchymal stem cells (Yin et al. 2021), the role of l-lactate in the regulation of a glycemic-induced senescent phenotype should be particularly interesting. In type 2 diabetic patients, with a chronical hyperglycemic state, (Brouwers et al. 2015; Adelsmayr et al. 2012), l-lactate is a leading biomarker, so it is presumable that l-lactate should play a role as a regulator in the glycemic impact of the stress response.

Two different of fundamental routes are to be put in the spotlight to further elucidate the involvement of l-lactate in the aging process of the skin: (a) the role of l-lactate in bioenergetics and mitochondria biology; (b) the role of l-lactate in stem cell commitment in the skin and mesenchymal differentiation. Both functions are intertwined with ROS biology.

## l-lactate in skin physiology

### l-lactate and skin biology during aging: the immune mircoenvironment

Due to the fundamental role of l-lactate in the mitochondria-ROS signaling, and therefore in the aging process, skin differentiation should be affected by l-lactate turnover and metabolism. Actually, l-lactate participates in the complex milieu of skin cellular components by interacting with the interplay stromal cells-immune cells, for example by switching off the pro-inflammatory immune response and promoting tissue renewal and repair (Selleri et al. 2016; He et al. 2019). Human stromal cells from mesenchymal origin (MSCs) release l-lactate and induce a lactate-mediated reprogramming in dendritic cells, i.e., MSCs produce large amounts of l-lactate and cause the differentiation of monocytes to M2-macrophages, so acting as an immunomodulant molecule (Selleri et al. 2016). The role of M2-macrophages in aging and skin biology is intriguing, because these anti-inflammatory phenotypes are typically skewed from precursors in tissue repair mechanisms, which encompass the involvement of Th2 cytokines mediating an M2 programming of monocyte-to-dendritic cells and moreover of apoptotic events, then contributing in tissue renewal (Kim and Nair 2019). It is possible that l-lactate works as a switcher in the M1/M2 skewing process, to ensure the ability of monocyte to polarize, as this ability is reduced in advanced senescent phenotypes (Mahbub et al. 2012). The existence of an M2-milieu in the innate immunity of skin, promotes cell differentiation and survival. It is well known that MSCs have a multi-lineage differentiating pattern, in order to improve wound healing, but also recent data reported that MSCs are joined to M2-skewed macrophages and by the co-occurrence of a hypoxic microenvironment (Lee et al. 2016).

### Role of hypoxia and staminality

Aging in the skin can be caused by the loss of the hypoxia-inducible factor 1 alpha (HIF-1α) (Rezvani et al. 2011). In normal human diploid BJ fibroblasts, l-lactate promotes the role of HIF-1α in shifting mitochondria oxidative phosphorylation to glycolysis (Kozlov et al. 2020). HIF-1α is a leading regulator of glycolysis and is able to promote the expression of several genes involved in glucose uptake and metabolism, such as pyruvate dehydrogenase kinase (PDK, isozymes 1–3) and pyruvate kinase muscle isozyme 2 PKM2 (Prigione et al. 2014). Usually, HIF-1α is rapidly degraded following its genetic translation in normoxic conditions but l-lactate has the ability to stabilize HIF-1α in the cell, prolonging its action and therefore contribution in reducing the impact of the aging process. This ability, as observed in BJ human fibroblasts is promoted by l-lactate via a ROS signaling (Kozlov et al. 2020). Stabilizing HIF-1α in dermal fibroblasts leads to the enhancement of PDK1 and PKM2 proteins, whereas PDK1 and LDHA are particularly increased in hypoxic conditions (Kozlov et al. 2020). The glycolytic shift is not only a switching on/off on aerobic/anaerobic metabolism but relates mitochondria function and l-lactate to cell replication and stem cell biology. In this perspective, it is interesting to observe that c-myc promotes a state of high energy supply, fundamental for stem cell generation, in which further components such as the estrogen related receptor alpha (ERRα) and its major cofactor, peroxisome proliferator-activator receptor gamma coactivator 1-beta (PGC1-β), are also involved (Prieto et al. 2018; Kida et al. 2015). Furthermore, in early somatic cell reprogramming, a fundamental role is exerted by the snail family of transcriptional repressor (SNAIL1), which has a role in the epithelial-to-mesenchymal (EMT) transition (Unternaehrer et al. 2014). l-lactate increase the expression of c-myc and SNAIL in dermal fibroblasts, so showing the crucial role of this catabolite as a signaling molecule in stem cell reprogramming (Kozlov et al. 2020).

The relationship between aging and MSCs has been recently reviewed (Liu et al. 2020a, b, c). In aged and senescent MSCs a down-regulation in the expression of C–C motif chemokine receptor 7 (CCR7), stromal cell-derived factor 1 (SDF-1) and its receptor chemokine receptor type 4 (CXCR4), and also of tumor necrosis factor receptor (TNFR) and IFN-γ receptor (IFNGR), have been observed (Liu et al. 2020a, b, c). Furthermore, an age-related decline in the gene expression of the runt-related transcription factor 2 (Runx2), the core binding factor α1 (CBFA1), and distal-less homeobox 5 (DIx5) as well as osteocalcin and collagen, has been reported, alongside with an increase in pro-adipogenetic components such as peroxisome proliferator-activated receptor-γ (PPAR-γ) and adipocyte fatty acid-binding protein (aP2) (Jiang et al. 2008). A close relationship between Runx2 and HIF-1α occurs to promote angiogenic signals (Kwon et al. 2011). l-lactate, by stabilizing HIF-1α even in normoxic conditions, promote vascular endothelial growth factor (VEGF) production (Song et al. 2018). In this perspective, therefore, l-lactate may contribute in dermal vascularization, which is a major issue in skin aging. An age-related decrease in dermal vascularization, might be due to impairment in VEGF signaling via the delta-like ligand 4 (Dll4) and Jagged-1 (Jag-1) (Gunin et al. 2014).

**Fig. 1:**
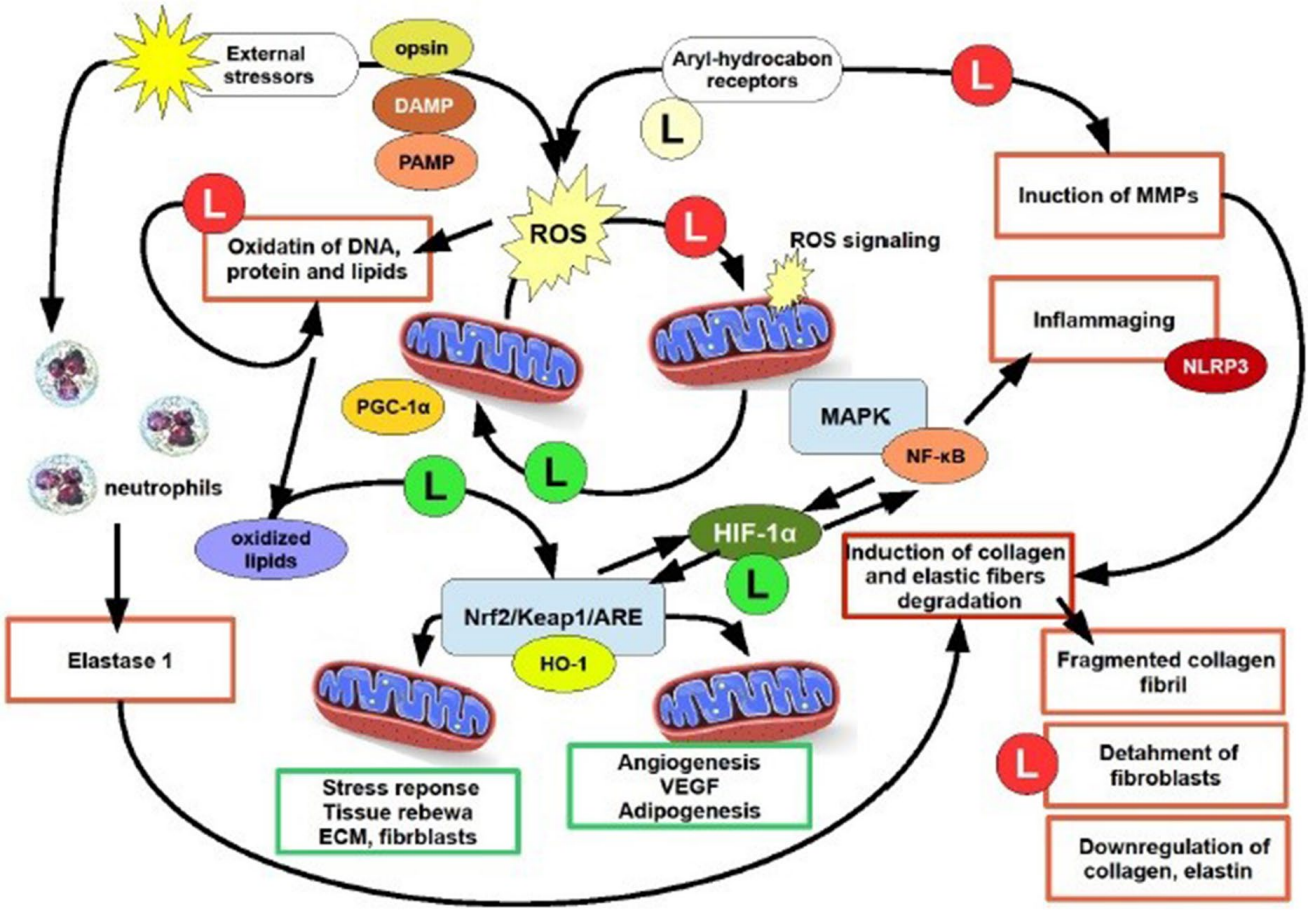
A schematic representation of l-lactate activity within the pathogenesis of premature/extrinsic skin aging. In the center of the cartoon is simplified the complex balance on ROS production by mitochondria, in order to assess ROS as signaling molecules. This mechanism allows mitochondria biogenesis, uncoupled events, mitokinesis and mitochondria fission/fusion, via the PGC-1α but l-lactate is also able to use oxidized lipids to trigger the Nrf2/keap1/ARE via mitohormetic mechanism. The activity by l-lactate on HIF-1α allows to bring together two quite opposite but interplaying pathways, i.e. the Nrf2/Keap1/ARE pathway with HO-1 and the MAPK/NF-κB pathway. Aging in the skin is exemplified by events within squares red lined squares, whereas events reverting the aging process and promoting survival and renewal are green lined squares. l-lactate is indicated as “l” within a green circle (if promoting or triggering), pale yellow if regulating, red if inhibiting. *DAMP* damage associated molecular pattern, *HO-1* heme oxygenase-1, *NF-kB* nuclear factor kappa‐B, NLR family pyrin domain containing 3, *PAMP* pathogen associated molecular pattern

Aging in skin can be considered a degenerating process starting from the increasing difficulty of mitochondria to ensure cells with the ability of promote survival process, stressors scavenging and stem cell/differentiation interplays at a balanced level. Figure 1 shows the possible relationship between l-lactate and mitochondria biology to elucidate the role of l-lactate in the aging process. The central core of this complex task is the ability of l-lactate to join metabolism and bioenergetic with the oscillating ability of mitochondria to regulate the cell fate. ROS are continuously to be adjusted to work as signaling molecules, whereas any excess must be buffered in order to prevent mitochondria stress and the impairment in their biogenesis and turnover. Further research should elucidate the role of l-lactate in this perspective.

## l-lactate, ROS and mitochondria: role in skin aging

### l-lactate and mitochondria

During aging, the role of mitochondria in skin physiology is particularly crucial (Stout and Birch-Machin 2019; Sreedhar et al. 2020). Many authors agree with the idea that aging involves immunity in a process known as “inflammaging” (Franceschi et al. 2007). Mitochondria are a sort of “powerhouses” of immunity, as mitochondrial DNA (mtDNA) may act as a danger-associated molecular pattern (DAMP) and the same outer membrane of these organelles act as a bench for signaling components such as RIG-1 and MAVS and can activate also NLRP3 (inflammasome) (Mills et al. 2017). Circulating mtDNA correlates with increased Body Mass Index (BMI) and aging (Padilla-Sánchez et al. 2020) and has been since years considered a major biomarker of inflammation and cancer (Yu 2012). A mitochondrial functional biomarker is therefore associated with the presence of mitochondria genomes in the cell. Actually, the mitochondrial DNA copy number (mt-DNA CN), an evaluation of the number of the organelle genomes per cell, is used as a functional biomarker associated with aging-related disorders (Longchamps et al. 2020). Furthermore, during aging mitochondria DNA mutations cumulate and respiratory function declines (Wei et al. 2009). Mitochondria functionality is so crucial for skin health during aging that some authors suggested very recently an artificial mitochondria transfer/transplant (AMT/T) to promote renewal of senescent skin cells, even revitalizing them (Balcázar et al. 2020).

The relationship between l-lactate and “mitochondria health” may be closely related to aging processes. Some cognitive and neurological disorders links mtDNA alterations with increase in circulating l-lactate (Valiente-Pallejà et al. 2020; Hanisch et al. 2006). The importance of l-lactate for mitochondria bioenergetics has emerged very recently; for example, Young and colleagues showed that l-lactate can support fueling mouse mitochondria energetics, in liver, heart and muscle, via mitochondrial LDH, further participating in ROS generation and in the production of H_2_O_2_ to an extent comparable to pyruvate (Young et al. 2020). If l-lactate is fundamental for mitochondria, the “shuttle hypothesis” formulated by Brooks some years ago may suggest that l-lactate is used also as a signaling molecule to connect bioenergetics with cellular turnover (Brooks 2009). This hypothesis may find a confirmation as l-lactate oxidized in mitochondria exceeds of 10–40% the oxidation of pyruvate for bioenergetics (Brooks 2009).

The role of l-lactate in mitochondria biology is fundamental at least because the metabolism of l-lactate occurs in mitochondria (Chen et al. 2016; Glancy et al. 2021). Moreover, the relationship between mitochondria and aging dates back to the sixties and is certainly a major issue to elucidate the possible role of l-lactate and mitochondria in skin aging (Rockstein and Brandt 1963; Sun et al. 2016). The majority of studies on the relationship between mitochondria dysfunction and mtDNA mutations leading to the aged phenotype have been obtained by the so called “mitochondrial mutator mouse”, a knock out laboratory animal a mutated D257A gene, a proofreading deficient form of the polymerase POLGg, acting on mtDNA.

In these mice the gene encoded by the nucleus is the only mtDNA polymerase, which, as mutated at the position 257, lacks of the proofreading ability. Mice with one or two copies of this mutated gene, accumulated a huge deal of deficient mitochondria and show an accelerated senescent phenotype respect to wild type (Kujoth et al. 2005; Trifunovic et al. 2004). So far, the relationship between aging, mitochondria and l-lactate, has been particularly highlighted in molecular neuroscience (Datta and Chakrabarti 2018) but it can be speculatively suggested that a major role of l-lactate in modulating and regulating mitochondria-addressed aging may be retrieved from many other tissue and organ models, such as skin, where the dynamics of mitochondria and their intracellular connections is fundamental (Mellem et al. 2017). In mitochondria biogenesis the transcription co-activator factor peroxisome proliferator-activated receptor-gamma coactivator-1 alpha (PGC-1α), is a master tuner of bioenergetics (Liang and Ward 2006). As a matter of fact, PGC-1α controls also l-lactate metabolism, for example by increasing the expression of MCT1, more than MCT2 and MCT4 in the skeletal muscle (Benton et al. 2008) and controlling the whole availability of l-lactate in the tissue (Summermatter et al. 2013). The administration of l-lactate increases the expression of PGC-1α, by inducing an enhancement in PGC-1α mRNA transcripts, and at the same time increases also pyruvate dehydrogenase kinase 4 (PDK4) and the mitochondrial uncoupling protein 3 (UCP3) gene expression (Kitaoka et al. 2016).

It is widely known that UCP3 should protect mitochondria from aging and also from inflammaging and fat-induced damage, via ROS as signaling molecules. Some authors, using C57Bl6 mice overexpressing skeletal muscle UCP3 (UCP3Tg), reported that 4-hydroxynonenal (4-HNE), an α-β unsaturated hydroxy-alchenal from lipidic peroxidation, dampens the age-related increase in ROS, due to the increased state IV of oxidative respiration from mitochondria by the UCP3 overexpression (Nabben et al. 2008). It is intriguing that 4-HNE is the major byproduct by oxygen-ozone treatment, which has been recently used even to improve the anti-inflammatory therapy against COVID-19 (Chirumbolo et al. 2021). The relationship between PGC-1α and ROS in particularly intriguing during the biogenesis of new mitochondria and mitophagy, where PGC-1α buffers the excess of ROS by eliciting the production of anti-oxidant scavenging enzymes (Baldelli et al. 2014). Mitochondria biogenesis and mitophagy are fundamental processes in counteracting aging (Wei et al. 2021; Bakula and Scheibye-Knudsen 2020; Chen et al. 2020). In this perspective, l-lactate may even have a crucial role.

The dynamin related protein 1 (DRP1), which regulates mitochondria biogenesis and mitophagy, i.e., mitochondrial and peroxisome fission, regulates also endoplasmic reticulum-generated droplets in the adipose tissue, so correlating the correct lipid storage in adipocytes with the mitochondrial survival (Li et al. 2020). Recent data on lung cancer cell models, reported that DRP1 promotes l-lactate utilization and suppresses oxidative stress from ROS (Hu et al. 2020). Targeting DRP1 allows to reducing the expression of heart shock proteins (hsp90) and increasing the ROS-mediated cleavage of hsp90, a mechanism that in turn will inhibit the MAPK and PI3K-mediated pathways, the ability of cell to use l-lactate and subsequently the ROS-mediated cell death (li et al. 2020).

Mitochondria fusion and fission are remarkable mechanisms in the development of the aging process, in which l-lactate would play a role (Liu et al. 2020a, b, c). Mitofusin-2, a protein of the outer membrane involved in mitochondria fusion, when deficient might regulate an adaptive response involving the increase in PGC-1α and in the transcription factor TFAM, to prevent mtDNA depletion (Kawalec et al. 2015). The bulk of evidence relating l-lactate with mitochondria and ROS strongly suggests that l-lactate, therefore, is intertwined with mitochondria turn over and probably with the molecular mechanisms leading to aging.

### Mitochondria, l-lactate and aged skin

The role of mitochondria in skin aging has been recently reviewed (Stout and Birch-Machin 2019; Hudson et al. 2016). Noteworthy, mitochondrial metabolism is a leading process for keratinocyte differentiation. Deletion of TFAM at the level of basal cells of epidermis causes a loss in ROS signaling (reduction in the mitochondria production of ROS) and therefore an impairment in epidermal differentiation and also hair growth (Hamanaka and Chandel 2013). ROS are fundamental elements of tissue renewal and differentiation in the skin, including dermal compartments and adipose tissue (Rigotti and Chirumbolo 2019). Therefore, if ROS controls many fundamental aspects of skin biology, l-lactate, which is intertwined with the mitochondria-ROS signaling machinery, is of the utmost interest for skin differentiation and aging.

If mitochondria play an utmost role in skin differentiation, their involvement in aged skin should be particularly crucial.

Aged skin shows marked deterioration in mitochondria structure, cristae numbers, mtDNA copy number and mutations and defects in mitochondria biogenesis, fission and fusion, a process observed in many other epithelial tissues (Schneider et al. 2020). Recent reports showed that ubiquinol (reduced coenzyme Q10) has a powerful anti-aging effect on human dermal fibroblasts (Marcheggiani et al. 2021) and is a metabolic resuscitator in post-cardiac arrest (Holmberg et al. 2021). The production od ROS is fundamental for propagating both Notch and β-catenin, signals that are very strategic for epidermis differentiation and hair follicle development (Marcheggiani et al. 2021). Genetic defects in Coenzyme Q_10_ (CoQ_10_) biosynthetic pathway may affect l-lactate regulation, as the CoQ_10_ deficiency, which may be caused by a homozygous stop variant in *COQ9* c.730C > T, pArg244*, should lead to neonatal lactic acidosis, general development delay and intractable seizures (Duncan et al. 2009).

As noted earlier, l-lactate protects skin fibroblasts via mito-hormesis (Zelenka et al. 2015). Previous literature has reported that l-lactate treatment upregulates the production of ROS as signaling molecules (Hashimoto et al. 2007), activates mitochondrial biogenesis via PGC-1α (Roland et al. 2014; Lezi et al. 2013) and stabilizes HIF-1α (Sonveaux et al. 2012). During aging, skin undergoes a complex plethora of biochemical and structural changes. For example, the role of NAD^+^-dependent deacetylases, known as sirtuins (SIRT 1–7), is fundamental to comprehend skin aging, particularly for photoaging, and the involvement of mitochondria biology (Su et al. 2020). Sirtuins 3 (SIRT3), 4 (SIRT4) and 5 (SIRT5), which are localized in mitochondria, are implicated in aging, besides to oxidative stress response and caloric restrictions (Gambini et al. 2011). Particularly for SIRT3 and SIRT4, their role is crucial for keratinocyte differentiation and wound repair (Su et al. 2020). Particularly for SIRT3, recent studies have reported that this deacetylase is a shield against aging and mitochondrial meltdown (Kincaid and Bossy-Wetzel 2013). Recent data reported that SIRT3 controls cell proliferation and glucose uptake overexpression (Cui et al. 2015). The majority of evidence regarding the role of sirtuins in the interplay cell metabolism-cell survival and proliferation/differentiation come from cancer studies (Gaál and Csernoch 2020), but SIRT3 is downregulated in lung idiopathic pneumonia with fibrosis (Rehan et al. 2021), where l-lactate is particularly elevated (Kottmann et al. 2012). It is possible to speculate that l-lactate is an alarming signal of a downregulation or impairment in the correct mitochondrial function and ROS availability as signaling molecules.

l-lactate, which is a molecular and metabolic starter to produce ROS as signaling molecules, is able to induce collagen synthesis and VEGF in endothelial cells (Zieker et al. 2009). Its action of collagen has been investigated in the past, when some authors discovered that l-lactate is one of the most powerful activator of collagen formation. By investigating L-929 fibroblasts Comstock and Udenfriend (1970) provided evidence confirmed by others with myofibroblasts and acetaldehyde, that l-lactate caused a significant (p < 0.02) enhancement in intracellular proline pool and collagen synthesis (Savolainen et al. 1984).

A fundamental role of l-lactate in reverting the aging process and promote the survival machinery of genes leading to tissue renewal, might be exerted on HO-1. This fundamentally occurs because HO-1, particularly skeletal HO-1, controls aerobic capacity, i.e. that the deletion in mice of the muscle-specific HO-1, in the transgenic mouse Tam-Cre-HSA-Hmox1fl/fl, changes the rate type IIA to type IIB muscle fibers, alongside with an overall disruption of mitochondria (Alves de Souza et al. 2021).

The induction of HO-1 under oxidative stress is mainly activated by the transcription factor nuclear factor erythroid 2-related factor 2 (Nrf2), which is regulated by the mitogen-activated protein kinase (MAPK), phosphoinositide 3-kinase (PI3k)/Akt, and protein kinase C (PKC) signaling pathways (Feng et al. 2017).

## Conclusions

Aging in the skin is a major process where the activity of l-lactate as a signaling molecule might provide biomedical research with heuristically valid insights, particularly about the role of this metabolite in lowering senescence in the skin and to elucidate further the mechanisms underlying skin aging.

In this review we have fundamentally highlighted that:

- l-lactate is a major signaling molecule able to interact with mitochondria biogenesis and turnover and tune the ability of cells to respond to stressors;
- The fundamental way by which l-lactate works as a signaling molecule involve mechanisms known as “mito-hormesis”
- Despite its nature of apparently useless byproduct, l-lactate plays fundamental roles in signaling of brain development related with exercise and in immune regulation;
- Its role in skin rejuvenation and aging control can be supported by the survival machinery led by mitochondrial biogenesis.

The role of l-lactate should be included in the wider role of small molecules working as signaling compounds, able to finely regulate cell survival, cycle and development. In this sense, the importance to deepen the activity and the biological meaning of this glycolysis byproduct is paramount.

## Data Availability

Not applicable. No data are reported in this manuscript.
